# Case Report: Safety and Efficacy of Omalizumab in a 13-Year-Old Patient With Chronic Spontaneous Urticaria and Type 1 Diabetes

**DOI:** 10.3389/fimmu.2022.853561

**Published:** 2022-04-12

**Authors:** Paolo Del Barba, Federica Del Tedesco, Giulio Frontino, Maria Pia Guarneri, Riccardo Bonfanti, Graziano Barera

**Affiliations:** ^1^ Department of Pediatrics, Istituto di Ricovero e Cura a Carattere Scientifico (IRCCS) San Raffaele Scientific Institute, Milano, Italy; ^2^ School of Medicine, Università Vita Salute San Raffaele, Milano, Italy; ^3^ Diabetes Research Institute, Istituto di Ricovero e Cura a Carattere Scientifico (IRCCS) San Raffaele Hospital, Milano, Italy

**Keywords:** chronic spontaneous urticaria, type 1 diabetes, omalizumab, pediatrics, glycemic

## Abstract

Chronic urticaria (CU) is defined by the presence of itchy wheals, sometimes accompanied by angioedema, lasting for at least 6 weeks. CU is treated with second-generation antihistamines, increased up to four times the normal doses for second-line treatment. Omalizumab (a monoclonal antibody anti-IgE) may be recommended as third-line therapy in children aged over 12 years. Few reports have suggested that glucose homeostasis is impaired in some type 2 diabetic patients receiving omalizumab, and even in non-diabetic patients, fasting blood glucose and HOMA-IR values appeared to be significantly increased. We report the case of a 13-year-old girl with diabetes mellitus type 1 and chronic spontaneous urticaria (CSU) refractory to standard recommended therapy that we treated with omalizumab at a standard recommended dose of 300 mg every 4 weeks. We observed a rapid and complete remission of CSU after treatment with this humanized monoclonal antibody without detrimental effects on the patient’s glucose control especially in terms of HbA1c (glycated hemoglobin), time in glycemic range (TIR), and daily insulin needs.

## Introduction

Chronic urticaria is defined by itchy hives and/or angioedema that recur for at least 6 weeks. It affects 0.1%–0.3% of children. When no specific eliciting trigger is identified, this condition is classified as CSU (1). It is believed that up to 50% of cases are associated with pathogenic autoantibodies most frequently related to thyroiditis and celiac disease (2, 3). It is known that children with T1DM compared with children without T1DM have an increased risk of urticaria (4).

Antihistamines are the first-line treatment for CSU, and if the standard dose is not effective, the dosage can be increased fourfold (5). Unfortunately, 25%–50% of patients do not respond to this treatment regimen and require other therapies, including antileukotrienes, cyclosporine, and more recently omalizumab.

Omalizumab is a recombinant humanized monoclonal anti-IgE antibody that binds free serum IgE, prevents its attachment to the high-affinity receptor on mast cells (FcεRI), and decreases receptor expression. Omalizumab was approved by the US Food and Drug Administration and the European Medicines Agency for antihistamine-refractory patients with CSU who are at least 12 years of age and significantly reduces the signs, symptoms, and burden of CSU (1).

In many studies, omalizumab has been demonstrated to be effective against CSU in children, even under 12 years of age (6–8).

Few reports have suggested that glucose homeostasis is impaired in some type 2 diabetic patients receiving omalizumab, and even in non-diabetic patients, fasting blood glucose and HOMA-IR values appeared significantly increased (9, 10). Another recent study demonstrated the safety of omalizumab in an 8-year-old child with type 1 diabetes mellitus (T1DM) and autoimmune thyroiditis (11).

## Case Description

A 13-year-old girl with T1DM was admitted to our hospital for poor glucose control under multiple daily insulin injections.

A review of her medical history and physical examination at presentation suggested that the girl had been suffering from chronic spontaneous urticaria unresponsive to antihistamine therapy. T1DM was diagnosed at the age of 11, appropriate treatment was initiated (multiple daily insulin injection therapy), and other type 1 diabetes-associated autoimmune diseases (celiac disease, autoimmune thyroiditis, and autoimmune atrophic gastritis) were excluded at diagnosis and yearly follow-up. Two years after the diagnosis of T1DM, she developed severe symptoms of chronic urticaria. Treatment with cetirizine (10 mg/day) was initiated, although no significant response was observed. There was no apparent correlation with exposure to common allergens nor physical stimuli. No history of allergy was reported; in particular, she did not report symptoms of oculorhinitis, asthma, or atopic dermatitis. She only referred one episode of urticaria in early childhood after taking amoxicillin-clavulanic acid.

The timing of onset and the frequency and duration of the hives were not consistent with insulin allergy (12, 13). Skin tests with insulin were not performed due to the persistence of hives and ongoing antihistamine treatment and the absence of concrete evidence supporting insulin skin test reliability (14).

Laboratory work-up revealed normal complete blood count and leukocyte formula (mild eosinophilia was detected: 600/mmc, n.v. 0–450) and normal serum total IgE. Specific IgE against common food and inhalant allergens showed weak positivity for only dust mites and ragweed. Liver, kidney, and thyroid function were normal; thyroid autoimmunity (anti-thyroid peroxidase, 11 IU/ml; anti-thyroglobulin, 31 IU/ml; and anti-TSH receptor, <0.8 IU/L) was negative. Celiac disease was also excluded, and inflammatory markers (CRP, ESR) were negative. Screening for other autoantibodies most frequently associated with CSU (ANA and ENA) showed negative results, with the exception of antinuclear antibodies (titer, 1:160). Complement fractions (C3, 1.13 g/L; C4, 0.22 g/L) and immunoglobulin classes were normal. Microbiological tests (including fecal *Helicobacter pylori* antigen) and stool parasite tests yielded negative results. Streptococcal infection was excluded. Urinalysis was also normal.

Due to treatment failure on standard cetirizine prescription, the dose was increased twofold, with only minimal improvement. We monitored the disease course on a weekly basis with a mean Urticaria Activity Score (UAS) of 36.

**Figure 1:**
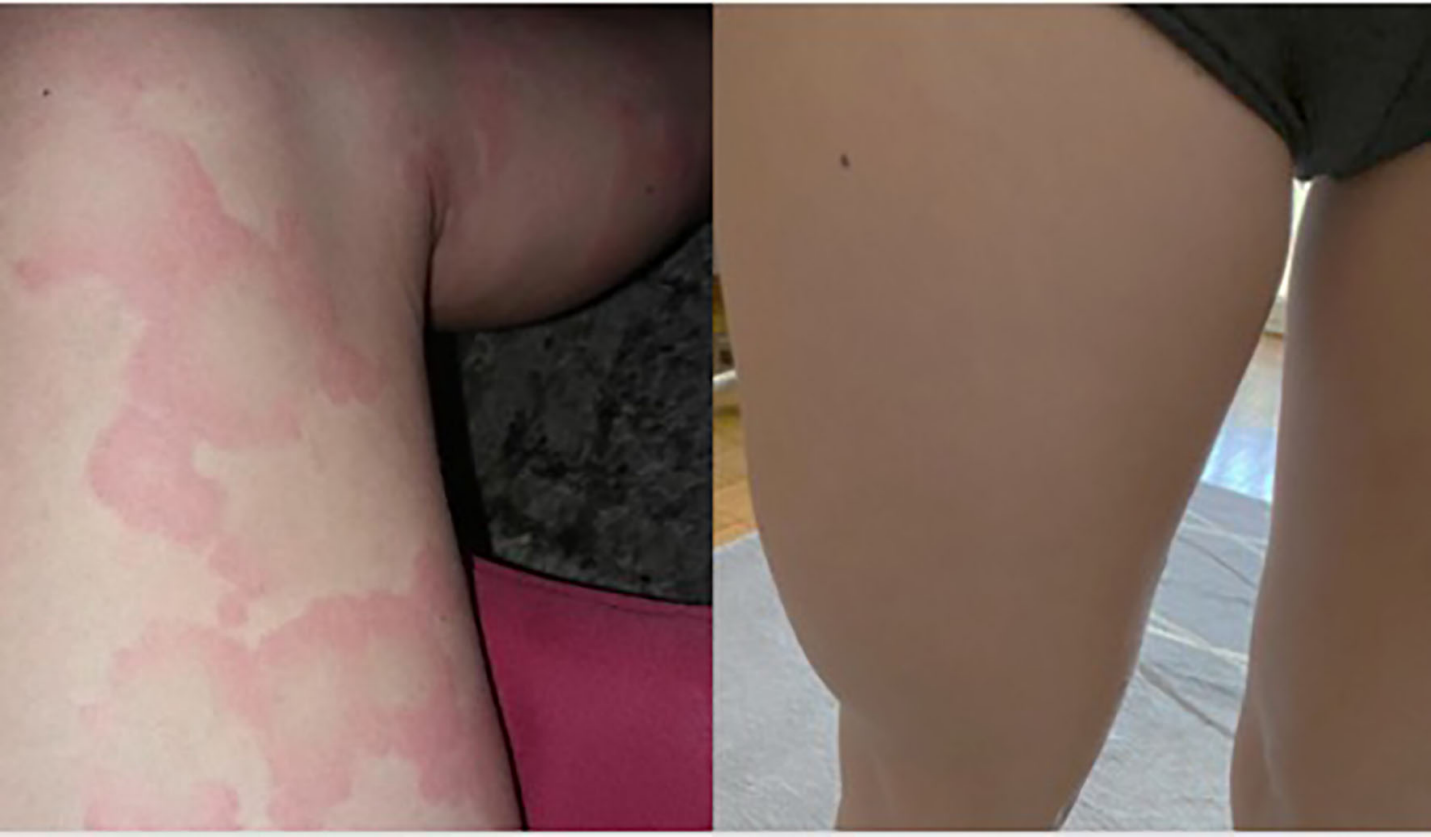
Cutaneous symptoms before and after treatment with omalizumab.

Given the persistence of clinical symptoms and the patient’s poor quality of life, omalizumab was started at a standard recommended dose of 300 mg intramuscular injection every 4 weeks. After the third dose and concomitant oral antihistamine treatment, symptoms significantly improved (UAS, 14), and only small urticarial elements remained on the extremities. After the sixth dose, CSU symptoms completely subsided, and antihistamine treatment was suspended (**Figure 1**, picture before and after treatment). Treatment was completed after 11 administrations of omalizumab with no relapsing CSU to date.

Glucometric data derived by flash glucose monitoring during treatment is shown in **Table 1**. Time spent in specific glucose ranges at baseline and after treatment were compared by chi-squared test. From baseline, time below range (TBR) has shown a slight but statistically significant reduction. However, this improvement has been apparently at the expense of an increased time above range (TAR) of 180–250 mg/dl. Time in range (TIR) did not vary. Baseline and post-treatment HbA1c were 6.8% and 6.9%, respectively, showing a substantial stable average glucose control during the treatment period. Daily insulin doses also did not vary significantly and were 1.1 U/kg at baseline and 0.9 U/kg post-treatment.

**Table 1:** Glucometric data derived by flash glucose monitoring during treatment.

	PRE (N = 2,656)	POST (N = 2,590)	p-value
TAR >250 mg/dl (%)	8	9	0.1940
TAR 180–250 mg/dl (%)	20	25	<0.05
TIR 70–180 mg/dl (%)	63	61	0.1357
TBR 54–70 mg/dl (%)	6	4	<0.05
TBR <54 mg/dl (%)	3	1	<0.05

## Discussion

Omalizumab is known to be an effective drug against chronic urticaria in children from 12 years of age. This monoclonal antibody has already proved to be beneficial for the treatment of severe asthma and CSU even when associated with other autoimmune diseases (15, 16).

Our case represents one of the few children with CSU in association with isolated type 1 diabetes who have been treated successfully and safely with omalizumab to date. Contrary to previous large-scale studies that suggested an increase in insulin resistance, assessment of flash glucose monitoring data in our patient suggests that treatment with this humanized monoclonal antibody did not determine detrimental effects on the patient’s glucose control especially in terms of HbA1c, TIR, and daily insulin needs (9, 10, 17). Further randomized controlled studies of CSU in pediatric patients with T1DM are warranted in order to more clearly assess the impact of omalizumab treatment on glucometrics and insulin resistance in this particular population. This is of particular concern especially for patients undergoing multiple daily insulin injections, as the ever increasing popularity of hybrid closed loops systems may prevent any omalizumab-related increased glucose variability.

## Data Availability Statement

The original contributions presented in the study are included in the article/supplementary material. Further inquiries can be directed to the corresponding author.

## Ethics Statement

Written informed consent was obtained from the minor’s legal guardian for the publication of any potentially identifiable images or data included in this article.

## Author Contributions

PB, FT, GF and MG conceived the idea of the clinical case and wrote the manuscript. RB and GB supervised the findings of this work. All authors contributed to the article and approved the submitted version.

## Conflict of Interest

The authors declare that the research was conducted in the absence of any commercial or financial relationships that could be construed as a potential conflict of interest.

## Publisher’s Note

All claims expressed in this article are solely those of the authors and do not necessarily represent those of their affiliated organizations, or those of the publisher, the editors and the reviewers. Any product that may be evaluated in this article, or claim that may be made by its manufacturer, is not guaranteed or endorsed by the publisher.
